# From housing to home and belonging: structural violence, ontological (in)security and women's mental health

**DOI:** 10.3389/fsoc.2026.1612133

**Published:** 2026-03-09

**Authors:** Roxanne C. Keynejad, Francisca Gaifém, Ivana Nikolić, Sewhareg Belay, Hiwot Hailu Amare, Hiwot Abera, Maria-Christine Mautner, Hanne Ochieng Lichtwarck, Frode Eick

**Affiliations:** 1 Health Service and Population Research, Institute of Psychiatry, Psychology & Neuroscience, King's College London, London, United Kingdom; 2 Department of Clinical Medicine, Aarhus University, Aarhus, Denmark; 3 Department of International Law, University of Belgrade, Belgrade, Serbia; 4 School of Public Health, College of Medicine and Health Sciences, Hawassa University, Hawassa, Ethiopia; 5 School of Public Health, College of Medicine and Health Sciences, Dilla University, Dilla, Ethiopia; 6 Internal Medicine III, Endocrinology, Metabolism and Gender Medicine, Medical University of Vienna, Vienna, Austria; 7 International Development, University of Vienna, Vienna, Austria; 8 Department of Community Medicine and Global Health, University of Oslo, Oslo, Norway; 9 Lovisenberg Diaconal University College, Oslo, Norway

**Keywords:** belonging, home, housing, ontological security, ontological insecurity, structural violence, women's mental health

## Abstract

Women's mental health is profoundly shaped by structural violence through housing, which impacts their experience of home and sense of belonging. This Perspective advances an integrative sociological framework across a continuum from the most concrete manifestation of shelter (housing) through symbolic representations of home, to the psychological experience of belonging to a place. We synthesize evidence for relationships between unstable, unsafe housing, abusive homes, social dislocation, and mental ill-health. Drawing on interdisciplinary scholarship, we specify how housing insecurity erodes community, relationships, and safety, generating ontological insecurity that amplifies vulnerability to interpersonal violence. We show that these dynamics are bidirectional: mental health conditions can impede access to resources and secure housing, reinforcing insecurity, while housing adversity is associated with worsened mental health. An intersectional lens reveals that minoritised women experience compounded harm through migration status, precarity, and policy regimes that limit protection and support. Our framework connects macro-level structures (national and international policy), meso-level institutions (welfare, health, and housing services), and micro-level lived experience, demonstrating how structural conditions become embodied as mental health symptoms. This Perspective situates ontological (in)security as central to the relationships between shelter, place, and women's mental health, identifying actionable leverage points for change. We argue for gender-sensitive, integrated interventions that expand access to safe, stable housing, co-locate mental healthcare with rights-based and anti-discrimination services, and address feedback loops between shelter, place, and mental health. By foregrounding housing as a key conduit of structural violence, this Perspective advances sociological understanding of how belonging and mental health are co-constituted and offers directions for research and policy to improve women's health and flourishing.

## Introduction

*I thought how unpleasant it is to be locked out; and I thought how it is worse perhaps to be locked in*.

Virgina Woolf

Although the human need for shelter is one of the most basic, the depth of psychological, social, and interpersonal significance of home and belonging to mental health is rarely considered or addressed. Woolf's epigraph captures the complexity of literal and symbolic meanings which home and belonging have for diverse women across cultures and spatio-temporal contexts. “Home” holds power as a refuge from political and societal threats, and Woolf's “room of one's own” can offer “a transitional space” in which women may separate from social expectations and chart an autonomous path (Alexander, 2000, p. 287). Equally, however, home can be a place of women's oppression or violence, as expressed here by Woolf.

This Perspective is grounded in experience of clinical mental healthcare and seeks to integrate interdisciplinary perspectives to elicit understanding with practical applications. We propose a framework for recognizing the gendered influence of shelter and place on women's mental health, integrating interdisciplinary perspectives from psychiatry, psychology, sociology and anthropology. We propose a framework situated across a continuum from the most concrete manifestation of shelter (housing), through the more symbolic concept (home), to the most psychological construct (belonging). We approach these three over-arching facets of shelter and place as lenses through which to integrate interdisciplinary evidence for positive and negative feedback loops involving structural violence/social protection, ontological in/security and women's mental (ill-)health.

We begin by situating our Perspective within the concept of structural violence. We then consider relationships between women's mental health and the three facets of shelter and place, moving from housing, through home, to belonging. When considering tangible access to housing, we review evidence for the mental health impacts of homelessness and “temporary” accommodation on women. We next consider how violations of the symbolic meaning of home through domestic abuse and displacement impact women's mental health, introducing the central concept of ontological (in)security. We then discuss how subversion of women's psychological experience of belonging by discrimination, migration, and gendered expectations impacts mental health. Finally, we unify the evidence through an integrative framework and discuss its implications for future research and practice, in times of evolving global instability.

## Gender dimensions of structural violence

The concept of structural violence is highly relevant to women's mental health, but not always familiar to clinicians. First coined by Johan Galtung, structural violence refers to often-unnoticed systemic inequities intrinsic to social structures at local, national, and international levels, which hinder people's ability to achieve their full potential (Galtung, 1969). Medical anthropologist Paul Farmer developed the concept, arguing that these inequities produce vulnerabilities in affected populations, enacting violence through harm and injury Farmer et al., (2006), including poor health. Using examples from Human Immunodeficiency Virus (HIV) care, Farmer et al. (2006) illustrated how the health impacts of social forces such as housing, poverty and racism are readily apparent to clinicians in practice, but lacking in biomedically dominated training and research.

Structural violence has been characterized as the “invisible social machinery of inequality that reproduces social relations of exclusion and marginalization” (Scheper-Hughes, 2004, p. 13). This concept explains the pervasive impact of demographic, economic, neighborhood, environmental, and socio-cultural factors (Lund et al., 2018): “social determinants” on health (Marmot, 2005). Differential exposure to this range of social determinants arises from social stratification: not only by social class, occupation, education or income, but also through intersecting aspects of people's identities, which confer the benefits of privilege or the harms of oppression (Bell, 2017). The unequal access to prevention, care, treatment, education, resources, and opportunities which arises from structural violence becomes embodied as health inequities, cyclically exacerbated by structural barriers to healthcare and other remedies (Fjelltorp-Veland et al., 2025; Hamed et al., 2020).

These cyclical relationships between inequity, adversity and health are especially apparent in mental healthcare, where the effects of structural violence on relapse and recovery are often marked (Alegría et al., 2023). For example, a study of 300 adults with severe mental health conditions in the United States found that adversities of home environment, social and economic circumstances interacted with gender to mediate recovery (Compton et al., 2020). However, the influence of such inequities can be compounded by the structures intrinsic to many mental health services, which normalize forms of violence, such as compulsory hospitalization and restraint.

Gender norms are complex, context-specific, and intersect with other social factors to influence the health of women and men across the life course (Weber et al., 2019). Although gender inequity, restrictive gender norms, and other forms of discrimination are social determinants of health (Heise et al., 2019), most psychiatric research has until recently been conducted without a gendered lens (Howard et al., 2017). Gender differences in the prevalence of mental health conditions are well-documented, worldwide, including among those with minoritised identities (Newcomb et al., 2020). Analyses of World Mental Health Survey data from 15 countries found elevated mood and anxiety disorders (odds ratios: 1.3 to 2.6) among women, relative to men (Seedat et al., 2009). The authors examined gender role traditionality, through female workforce representation, female education, median marital age and birth control. Reductions in gender role traditionality were associated with narrowing of gender differences in depression across countries, suggesting that social structures driving gender inequity impact the prevalence of mental health conditions.

The psychosocial pathways model (Stansfield and Bell, 2019) offers a framework for understanding how structural violence and its risk factors interact. Synthesizing findings of the World Health Organization (WHO) commission on the social determinants of health and related initiatives, as well as evidence syntheses and expert consensus, the psychosocial pathways model provides a visualization of the relationships between social determinants, psycho-social factors, health behaviors, physical and mental health outcomes. A key insight is how the effects of social determinants on health are mediated by psycho-social pathways, such as stress, control, self-efficacy, resilience, relationships, social cohesion and social capital. The model shows that integrating interdisciplinary understanding of how social determinants interact with psychological processes to influence mental health outcomes can illuminate complexity, to inform research, policy and practice. The psychosocial pathways model provides a specific example of Farmer et al. (2006)'s contention that linking public health activities to structural interventions can provoke “virtuous social cycles”. In the following sections, we consider the evidence for vicious and virtuous cycles between women's mental health and three facets of shelter and place: housing, home and belonging.

## Housing

Structural violence is perpetuated through lack or violation of social and economic rights to the fulfillment of basic needs (Farmer et al., 2006). International law affirms the right to adequate housing (article 11.1; UN General Assembly, 1989) and provides a common legal consensus on key aspects of this right. However, national laws often fail to address the full scope of housing difficulties, enabling commodification, precarity, and homelessness (UN General Assembly, 2019a). Structural violence is compounded by the fact that safe, secure, affordable housing is not uniformly distributed. Groups minoritised in terms of ethnicity, sexuality, gender identity, disability, refugee and sole caregiver status disproportionately experience unstable and poor quality housing, homelessness and negative treatment by services (Shelter Scotland, 2024). Homelessness and temporary accommodation are two examples of housing adversity with established mental health impacts, the gender dimensions of which are often overlooked.

### Homelessness

Pathways to homelessness are gendered. In many countries, women lack equal rights to land and property ownership, which may depend on their relationship with a male relative (UN General Assembly, 2019a). When relationships end, women may face eviction, often becoming “hidden homeless:” staying transiently with a series of successive friends and family.

Mental health conditions and substance use disorders are prevalent in homeless, populations, although women are proportionally under-represented in research studies (Gutwinski et al., 2021). Homeless women appear to have a higher prevalence of mental ill-health and past trauma than homeless men (Milaney et al., 2020; Power, 2023). Furthermore, social isolation and traumatic experiences while homeless can exacerbate existing mental health conditions and substance use, increasing vulnerability and further hindering women's ability to secure stable housing (Greene et al., 2024). A study of over 2,000 women in the United States found that housing instability and disarray but not material conditions were associated with depression and generalized anxiety disorder (Suglia et al., 2011). Thus, in high-income country settings, the uncertainty and disorganization of precarious housing may be more detrimental to mental health than specific housing conditions.

Less is known about the mental health of homeless people in low- and middle-income countries. Few studies have investigated severe mental health conditions, such as schizophrenia, bipolar disorder, and severe depression, among people who are homeless in these settings (Smartt et al., 2019). In Addis Ababa, Ethiopia, 90% of 217 street homeless adults had a mental health or substance use disorder (Fekadu et al., 2014). The 10% of the sample who were female reported high rates of sexual abuse, indicating the relevance of gendered traumatic experiences to women's experiences of homelessness. Although the needs of homeless people with severe mental health conditions in low and middle-income countries are beginning to receive research attention (Hanlon et al., 2025), a gendered lens is essential. Qualitative studies in Addis Ababa have identified how childhood trauma and sexual abuse (Yohannes, 2025), poverty, family and relationship instability predisposed women to homelessness and prevented their exit from it (Haile et al., 2020).

### Temporary accommodation

Although street homelessness is the most publicly visible form of housing adversity, precarious housing is extremely common. Despite high morbidity and mortality, more than 1.8 billion people live in informal settlements or inadequate housing, worldwide (UN Human Rights, 2025). Inadequate housing is also a gendered problem. In 80% of the 59 low and middle income countries providing data, women were overrepresented in urban slum areas disconnected from essential services (UN Habitat, 2020). Unsafe, insecure housing with limited community or police oversight and poor sanitation facilities was associated with gender-based violence victimization in Delhi and Nairobi (UN Habitat, 2020): a vicious cycle of structural violence.

Although informal settlements are more common in low and middle-income countries, lack of affordable accommodation in high-income countries means that many people with mental health conditions are housed “temporarily” for protracted periods (Shelter, 2025), with a range of adverse health impacts (Shelter, 2004). Although the quality of shelter and provision of essential infrastructure are central to adequate housing (UN General Assembly, 2011), temporary accommodation is rarely designed to address caring responsibilities, safety and security concerns, which disproportionately affect women (Shelter Scotland, 2024).

Housing insecurity entails continuous uncertainty, including the threat of eviction. A range of studies in high income countries has identified poor mental health as a risk factor and consequence of eviction, especially for low-income families (Clarke et al., 2017; Vásquez-Vera et al., 2017). In low and middle-income countries, forced eviction is associated with poor mental health (Richardson et al., 2016) and increased risk of sexual violence (Jelle et al., 2021). Following forced eviction, women may be relocated to transient camps or detention centers, where they report difficulty adjusting to poor conditions and feeling trapped, suffocated, dehumanized, ashamed, devalued, mistrustful, and isolated (Abdulrahman, 2025).

A broad literature supports associations between housing adversity, poor mental health and violence exposure among women in low, middle and high-income country contexts. In keeping with the psychosocial pathways model (Stansfield and Bell, 2019), protracted uncertainty and lack of control can both precipitate and perpetuate mental health conditions among women with insecure housing. Despite the importance of housing to mental health, relevant services work largely in isolation. The exceptions are homeless shelters responsive to addictions, and specialist mental health supported housing, which have been most widely studied in high income countries such as Canada (Barker et al., 2022; Kirst et al., 2020).

Farmer et al. (2006) described addiction as having been “desocialised”: “viewed as personal and psychological problems rather than societal ones” (p. 1690) which followed legacies of oppression, including genocide and slavery. Similarly, the commodification of housing by capitalist, individualistic societies allows its gender dimensions and relationship to mental health to be disregarded in favour of personal attribution of responsibility. Having discussed the relevance of tangible accommodation to women's mental health, we next consider the symbolic and emotional experience of home.

## Home

Adequate housing is necessary but not sufficient for mental health. For example, “housing first” initiatives provide accommodation to homeless persons without imposing any requirements on residents, such as abstinence from substances or medication concordance. Research in Canada found that provision of such housing did not, in isolation, improve homeless women's mental health (O'Campo et al., 2023). This finding supports other studies suggesting that beyond shelter, the sense of home offers women social, psychological, and cultural foundations (D'Alessandro and Appolloni, 2020) for mental health.

Influential feminist thinkers have long critiqued the valorising of home for women (Friedan, 1963), pointing to its weaponisation to perpetuate oppression. However, exclusive critique misses the value home can have for women's mental health. Young (2005) proposed four normative values of home: safety, individuation, privacy, and preservation. Young (2005) characterized home as “the site of the construction and reconstruction of one's self” (p. 181). Thus, while women's wellbeing can be nurtured by positive experiences of home, unsafe or threatening homes pose risks.

A central concept capturing the psychological benefits of home is that of ontological (in)security. Ontological insecurity captures feelings of vulnerability and instability arising from a lack of control over one's living conditions, including a lack of “biographical continuity that typically underpins confidence in life's stability” (p. 4; Manzo and Grove, 2024). Ontological security was coined by Laing (1960), who proposed that lacking a coherent sense of self drives distorted perceptions of reality in schizophrenia. More recently, ontological insecurity has been used to understand experiences of disturbed identity among people with emotionally unstable personality disorder (McDonald et al., 2010). Attachment theory proposed that infants explore new environments, using their primary caregiver as a secure base (Ainsworth, 1964). For adults, individuated from their attachment figure, a stable home base from which to explore a potentially threatening outside world may support a sense of personal coherence, affording ontological security.

A study which interviewed home owners in New Zealand identified four (context-specific) facets of ontological security (Dupuis and Thorns, 1998). These were consistent social and material surroundings, a space in which daily life is enacted, a safe place free from scrutiny and over which the person has control, and “a secure base around which identities are constructed” (p. 24). Ontological security highlights psychological mechanisms by which a stable home may confer mental health benefits.

In a further vicious cycle of structural inequity, ontological insecurity is associated with gendered forms of structural violence. A study which interviewed women in Ontario found that perceived ontological insecurity was influenced by gendered, trauma-associated housing adversity and harmful substance use (Perri et al., 2025). Similarly, feeling safe from sexual violence and exploitation were central to the benefits of a home for women with severe mental health conditions in New York (Padgett, 2007). In keeping with the psychosocial pathways model, ontological security provided by home has potential to further a virtuous cycle, benefitting mental health. However, the safety, stability, and security of home can be violated from within and without. Two examples with well-documented mental health impacts are domestic abuse (Devries et al., 2013) and displacement (Bendavid et al., 2021).

### Domestic abuse

Perhaps the most common violations of women's right to a safe and secure home are violence and abuse perpetrated in domestic settings. Across 201 studies enrolling over 250,000 women, the lifetime prevalence of intimate partner violence (IPV) victimization was 37%; past-year prevalence was 24% (White et al., 2024). The structural violence of patriarchal norms and women's economic, social, and political disempowerment are barriers to leaving abusive relationships, which further increase the prevalence of IPV. Many victim-survivors lack autonomous access to accommodation (Lacmanović and Nestorov, 2021), leaving them dependent on often limited voluntary sector or charitable provision. Meta-analyses of longitudinal studies have shown that IPV victimization is bidirectionally associated with depression, suicide attempts (Devries et al., 2013), and alcohol use (Devries et al., 2014). That is, IPV is associated with subsequent depression, suicide attempts and alcohol use *and* these conditions are themselves associated with subsequent IPV.

Although the terms IPV and domestic abuse are often used interchangeably, they have distinct definitions. Domestic abuse as defined in UK law encompasses harm caused by adult family members as well as partners and ex-partners (Home Office, 2013). People with severe mental health conditions have higher prevalence of domestic abuse victimization than the general population (Khalifeh et al., 2015), and can face additional barriers to help-seeking. Coercive control is a type of domestic abuse, now a criminal offense in its own right in the UK (Home Office, 2023). Coercive control comprises acts dominating the person, including removing means of independence and escape. Previously conceptualized as “intimate terrorism” (Stark, 2010), such abuse can “colonise” the mind of the victim, changing their behavior through anticipatory anxiety, impacting mental health through feelings of entrapment (Lohmann et al., 2024). This phenomenon enables threats, intimidation, traumatic memories and associations to continue to influence women's mental states after they leave the domestic setting.

Despite the term, domestic abuse can be perpetrated outside the home, including through online activity (Rogers et al., 2023). Relatively recently acknowledged, technology-facilitated abuse exploits digital developments to extend a perpetrator's reach beyond the home. While both men and women report technology-facilitated abuse victimization, its perpetration appears to be largely by men (Rogers et al., 2023). Tracking technology, online platforms and novel digital threats, often designed to shame or humiliate the victim for violating culturally mandated gender roles, mean that physically leaving does not afford escape. Digital coercive control is “spaceless,” with cyber-stalking and online harassment leaving women under constant surveillance. Despite its untethered nature, digital coercive control is still shaped by place and space, as it has different forms and consequences depending on women's geographical locations, be they isolated rural areas or readily accessible urban settings. This new form of domestic abuse is “transcending borders and boundaries in new ways” (Harris and Woodlock, 2019, p. 537), leaving women feeling unsafe online and offline.

As Harris and Woodlock (2019) outline, technology-facilitated abuse can be deeply private, leading women to police their own behavior and online activity in anticipation of surveillance. It can also be extremely visible, co-opting friends, family, and colleagues to shame women's public persona. The potential psychological impacts of such experiences might include hypervigilance, avoidance, compulsive checking, paranoia and self-scrutiny. The emerging field studying technology-facilitated abuse is yet to grapple with the full mental health implications of its psychodynamic complexities.

While relationships between violence, abuse, and mental health are a focus of research (Oram et al., 2022), the meaning given to home by women who experience these intersections has received less attention. Intrusion of fear and violence through domestic abuse into what should be a safe and sacred space has clear psychological effects. In Turkey, domestic violence survivors characterized home making, to create a “home feeling,” as a defense against their “unhomely” marital houses, enabling them to cope with the transition after leaving (Lordoglu, 2023). In Canada, female IPV survivors' understandings of “home” were grounded in ontological security, through safety, community, and comfort, as well as material stability (Woodhall-Melnik et al., 2017).

Adverse childhood experiences, the majority of which take place in the home, including maltreatment, witnessing domestic abuse, parental mental ill-health and substance use disorders are risk factors for a range of future mental health conditions (Lewis et al., 2019). Childhood abuse is also a risk factor for revictimisation in adulthood, by domestic or sexual violence (Coid et al., 2001), including among people with severe mental health conditions (Anderson et al., 2016). The authors found that both men and women with severe mental health conditions and a history of childhood abuse were at elevated risk of adulthood abuse, but the association was higher for women. Despite a wealth of evidence about the impacts of abuse on mental health, psychological associations with home, as an arena for abuse, a trigger for traumatic memories or a place of healing remain under-studied.

### Displacement

In contrast to the interpersonal impacts of domestic abuse on mental health, are the geopolitical forces driving women's displacement from their homes. Women, children and families are increasingly uprooted by events beyond their control, such as climate change and armed conflict (Simmons, 2023). These causes of displacement have mental health impacts independent of those resulting from the experience of being displaced. Damage and disruption to homes, communities and livelihoods by extreme weather events are detrimental to women's health (Charlson et al., 2021), as are heat exposure, air pollution, dietary deficiencies, and infectious diseases arising from climate change (Rothschild and Haase, 2023). Gender inequity exacerbates these harms (Lawrance et al., 2021), through compounding by food insecurity, forced migration, and loss of social networks caused by climate events (Goudet et al., 2024). Many such experiences entail the loss and destruction of ancestral lands. A systematic review identified adverse mental health impacts of land dispossession as well as increases in gender-based violence (Ninomiya et al., 2023).

Women's mental health and physical safety are impacted directly by conflict, and indirectly by displacement (Bendavid et al., 2021). Bereavement, injuries, and socio-economic hardship arising from conflict are associated with common mental disorders (depression, anxiety disorders, and post-traumatic stress disorder) and suicidal ideation (Woldetsadik et al., 2022). In addition, war places women at risk of conflict-related physical and sexual violence perpetrated by military personnel, combatants, other strangers, and intimate partners (Asefa et al., 2024; Hossain et al., 2014). Feeling undermined by subversion of traditional gender expectations and economic pressures may increase men's perpetration of IPV during turbulent times (Falb et al., 2014; Restrepo et al., 2024), exposing women to abuse inside and outside the home. A negative feedback loop has been proposed, whereby social unrest, violence, and conflict are fostered by reduced social cohesion and community participation, which arise from poor community mental health (Miller, 2023). A mixed methods study in Uganda identified additive effects of IPV and armed conflict on women's depressive symptoms (Mootz et al., 2019). A study in post-war districts of the Amhara region of Ethiopia found that anxiety and depression were the commonest consequences of violence against women (Asefa et al., 2024).

The long-lasting nature of many current conflicts, in urban settings directly affecting civilians, has increased women and children's exposure to traumatic experiences (Bendavid et al., 2021). Women and children comprise the majority of forcibly displaced persons, and for many, displacement becomes protracted. For people displaced long-term, the transitional state of *liminality* captures the experience of powerlessness and marginalization while exerting agency to create a home despite the many barriers to doing so (Brun and Fábos, 2015).

The adverse mental health impacts of armed conflict are also not confined to those directly affected. Accumulating individual and collective traumas also impact the day-to-day well-being and collective memory of diaspora communities overseas (Hirschberger, 2018). Diasporic collective memory is shaped by aspects of the new country of residence and evolving armed conflict in the country of origin (Karim and Baser, 2022). There is growing evidence that learning about conflict in one's home country through social and other media is associated with anxiety, depression and vicarious trauma among diaspora communities caught between two worlds (Gesesew et al., 2025).

Diasporic memory evolves continuously, reshaped and reinterpreted by each generation in light of new geopolitical events (Baser and Toivanen, 2024). The integration of past and present can be positive, mobilizing diasporic communities to aspire to alternative individual and collective futures (Müller-Funk et al., 2023). Aspirations can facilitate hope and strengthen emotional resources, to endure struggles in the present. However, feminist theory has highlighted the disproportionate emotional labor upon women to invoke the past and evoke the future. An anthropological study of Greek women who emigrated to English-speaking countries following the Second World War found that they performed substantial cultural labor, “sandwiched” between their parents' nostalgia and their children's distance (Tsolidis, 2011). Diaspora women's mental health can be impacted by tensions between patriarchal cultural legacies and xenophobia in their new home.

Brun and Fábos (2015) proposed a tripartite conception of home, as an idea and a practice, in contexts of protracted displacement. They distinguished “home” (daily tasks of home-making) from “Home” (values, traditions, memories, and feelings of home), and “HOME” (political, historical and institutional conceptions). This approach offers a framework for understanding how the complexity of displaced women's conceptions and experiences of home may “retreat, emerge, or reappear in different configurations over time” (p. 15) – and how this may help them to endure extended uncertainty. These strategies may explain psychological resilience and preserved mental health among many individuals living in states of profound uncertainty.

The concept of ontological (in)security and the examples of domestic abuse and displacement are instructive for understanding the cyclical impacts of structural violence on women's mental health. If housing is the most concrete manifestation of shelter and home the more symbolic, belonging captures psychological experiences conferred by attachment to community and place.

## Belonging

Place-making is the active process of building a relationship with a new (Lems, 2016) or familiar (Cresswell, 2014) location. This process is often described in diasporic studies as a dynamic, multi-layered practice, continually negotiated across place, time, and social relations (Hall, 2015; Yuval-Davis, 2006). Anthropologists moved, during “the spatial turn,” from viewing people against the backdrop of their geographical context to seeing place and space as forces which shape human experience. While this transition drew scholarly attention to displacement, the shift to viewing people as “boundless” (Appadurai, 1988) has been argued to neglect the importance of place to people's realities (Lems, 2016).

Similar to scholars identifying benefits of home in the face of its dismissal as a place of women's oppression, Lems (2016) challenged characterisations of putting down roots as being trapped in one place. She presented the case of Halima, a Somali woman in Australia. Rather than yearning for freedom, “her fear is the fear to remain in transit and unfitting forever, to never arrive anywhere, to never be someone in relation to somewhere anymore” (p. 333). Lems identified the existential importance of “being in place” and belonging to Halima's sense of self. Halima says “I don't want to be in transit. My heart is working hard to settle, to settle and to belong to this country” (p. 324). However, Yuval-Davis (2006) argues that belonging comprises not only a personal sense but also the public, institutional processes that draw boundaries, gatekeep, and decide who can belong, on what terms.

If belonging captures the ultimate sense of ontological security, migration, discrimination, and marginalization are forces of structural violence which threaten both, with impacts on women's mental health. Even in conditions of exclusion, othering and judgement, however, women identify home, community, and faith as routes to belonging and wellbeing.

### Migration

The importance of belonging is clear when considering the health and social adversities faced by the increasing numbers of people who migrate (WHO, 2023). Both the mobility and health implications of migration are highly gendered (Abubakar et al., 2018). Many women who migrate are exposed to violence and exploitation before, during and after the journey, leading to dependence on others in transit, and once settled in their destination.

Rooted in colonial legacies, border regimes shape inequities between those who migrate, through differing attitudes to citizenship and legal status (Damsa and Franko, 2023). Structural violence by racialised border regimes in destination countries (Freedman et al., 2022) includes efforts to constrain migrant women's reproductive autonomy (Bhatia, 2023) and may unintentionally exacerbate gender-based violence by placing women at greater risk (Reilly et al., 2022).

The mental health of undocumented migrant women is affected by experiences of existential displacement, leading to feelings of “embodied unbelonging” (Bendixsen, 2020). That is, state actions enacting the classification of women as “illegal” impact their health and bodily experience of “being-in-the-world” in their host nation, exacerbated by restricted access to healthcare and other rights. Depressive and anxiety symptoms are highly prevalent among undocumented migrants. In Norway, 86% of undocumented migrants (*n* = 90, 43% female), endorsed clinically significant mental health symptoms (Myhrvold and Småstuen, 2017). Precarity, fleeing war or persecution and histories of abuse were associated with distress. Anxiety about providing for dependents and exploitative practices meant that family and work were not protective. Structural violence also appears to contribute to increased perinatal mortality and adverse maternal outcomes among undocumented migrants (Eick et al., 2024; Misje et al., 2025), a group which already faces elevated prevalence of perinatal mental health conditions compared to the general population (Stevenson et al., 2023).

Even in settings with access to paid employment, many female migrant workers suffer from structural violence. Large numbers of women migrant care workers deliver and improve health services worldwide, without themselves receiving full social protections (WHO, 2017). They often face precarity, exploitation, and gender-based violence. The mental health impacts on female migrant workers (Habtamu et al., 2017) diminish opportunities to integrate into the new country, worsening alienation and isolation (European Network of Migrant Women, 2021). Women who migrate for care work enter a “global care chain”, often leaving their own children in the care of others (Nadasen, 2017), underlining inequities with their employers, and reinforcing their separation from home. Such experiences have established mental health impacts on female migrant workers (Vianello et al., 2024), such as depressive and anxiety symptoms (Jecker and Chin, 2019).

However, despite hardships and adversities, studies have identified a range of coping strategies for attaining a sense of belonging. Bendixsen (2020) found that “irregular” migrants in Norway combatted embodied unbelonging through acts of resistance and exertions of agency. Where available, they established their sense of belonging by being-in-the-world through faith, religious community, and friendship. There is growing acknowledgment that trauma-informed mental healthcare for migrant women must recognize these and other sources of strength and resilience (Babatunde-Sowole et al., 2020).

### Discrimination

While migration can impede women's sense of belonging in their host country, discrimination against those perceived as “other” targets citizens and immigrants alike, for arbitrary reasons which evolve with socio-political preoccupations. Othering is a fluid, shifting process enacted by dominant actors and institutions through discourse, representation and institutional practice. Othering groups social identities, with those who are minoritised being classified as alien (Canales, 2000; Horowitz, 1989, pp. 66-67). Potential means of othering which scholars have identified range from biopolitical categorization (Foucault, 2003), division between settlers and “natives” (Fanon, 1961) and homonationalism (Puar, 2018; Slootmaeckers, 2019) to hospital systems for undocumented migrants (Fjelltorp-Veland et al., 2025), multiculturalism (Equihua, 2017, pp. 19–20) and white feminism (Cavanaugh, 2023, pp. 284–285; Vergès, 2021; Zakaria, 2021). Othering may be considered the other side of the coin that is belonging, with different facets of social identity defined by contrast to what one is not (Bhugra et al., 2023). The resultant marginalization of those occupying any micro-identities classified as “other” threatens their sense of belonging, depending on the context.

Internalized othering of Black people by white colonizers was central to the pioneering psychoanalysis of Fanon (1952). The minority stress model encapsulates how internalized prejudice, negative societal attitudes, expectations and actual experiences of discrimination predispose minoritised groups to poor mental health (Meyer, 1995). The mental health impacts of racism are well-known, including intersections with sexism toward ethnically minoritised women (Vines et al., 2017). These harms are compounded by complex relationships between intergenerational and historical racial trauma, parental and child experiences of and reactions to discrimination. Cycles of disempowerment arising from structural and identity-driven exclusion are thought to drive excess psychosis among ethnic minority (Jongsma et al., 2021) and migrant groups (Hatch et al., 2016), via processes of alienation and socioeconomic disadvantage. These cycles are amplified by interactions between systems of inequality, such as race and gender-based discrimination (Nixon, 2019).

The routine othering of women is captured by Simone De Beauvoir (1949)'s characterization of man as “the subject”, “the absolute”, “the essential” and woman as “the Other.” Indeed, inclusion of women, including pregnant women, and reporting of sex and gender in medical research publications is sufficiently variable to merit a dedicated policy framework in the UK (Witt et al., 2023). White, able-bodied, majority language-speaking men are usually the template for the design of health and social care systems, services and research, which then inadvertently discriminate against minoritised service users (Criado Perez, 2019). Even in high-income countries, outside perinatal-specific mental healthcare, women routinely follow trauma-uninformed pathways designed for the needs of men (Saunders et al., 2023).

Lack of consideration of women's specific, holistic needs may perpetuate discrimination by increasing the use of coercive mental healthcare. For example, a meta-analysis found that associations between minoritised ethnicity and compulsory detention in psychiatric hospital were stronger in studies with larger proportions of female participants (Barnett et al., 2019). This finding has at least two possible drivers. People with intersecting marginalized identities (in this case, females of minoritised ethnicity) may experience more severe mental health crises than those with non-minoritised identities—perhaps in part due to increased structural violence exposure—increasing their need for involuntary admission. In addition, people with multiple minoritised identities might provoke greater concern within the social structures (invariably established by people with non-minoritised identities) which respond to illness episodes, such as police and emergency medical services, increasing the likelihood of compulsory hospitalization. To optimize mental healthcare for minoritised women and others, both factors require intervention.

Clustering of structural violence exposure means that people with mental health conditions may experience othering through a range of marginalized micro-identities. For those recovering from severe mental health conditions, belonging has been understood as connecting with others, affiliation, inclusion and integration (Doroud et al., 2018). The authors found that the concept of place played a central role in processes of being, doing, becoming and belonging during mental health recovery. Thus, while othering may both predispose women to mental ill-health and result from it, finding belonging can offer pathways to healing.

### Gendered expectations

A final means by which women's sense of belonging is threatened, is through norms and expectations of femininity and womanhood. The mental health of women with intersecting marginalized identities, such as those who sell sex, is impacted by inter-related, stigmatized vulnerabilities, to substance use (Lichtwarck et al., 2023), gender-based violence (Beattie et al., 2020), HIV infection (Shannon et al., 2014), and criminalization (Lyons et al., 2020). These intersecting forms of structural violence increase women's need to access health and social care but feeling “undesired by society” impacts women's ability to trust service providers (Lichtwarck et al., 2024).

For women violating social norms, the ontological security of a safe home has potential to provide a sense of control essential to rebuilding their life and work, toward a sense of belonging. A study of women leaving prison in New Zealand found high exposure to domestic abuse and substance use in their homes prior to and during offending (Low and Mills, 2025). Women identified their new homes, free from these adversities, as physical expressions of their new identity, which reinforced desistance from offending.

While motherhood can foster a sense of belonging or inclusion, difficult experiences and disappointed expectations can generate feelings of dissonance (Keynejad et al., 2023) and ontological insecurity. For example, qualitative studies have identified feelings of alienation among migrant women who had children in their host country, via isolation, powerlessness and contradictory role expectations (Yang et al., 2022).

As well as a time of major biological, psychological, and social adjustment and high socio-cultural expectations, the perinatal period is one of elevated mental health risk (Howard and Khalifeh, 2020). Women experiencing intersecting forms of structural violence and trauma alongside severe mental health conditions have more social care involvement in their family life (Lever Taylor et al., 2023). Cycles of family disruption can become established, with child removal associated with further structural violence (Broadhurst and Mason, 2020) as well as maternal mortality (De Backer et al., 2025).

Struggling with motherhood not wishing to become a mother, or having difficulty conceiving can provoke profound distress. However, social structures which offer a sense of wider belonging such as education, employment, income, health insurance, social support, and religion appear to be important protections (Bagade et al., 2023; Xie et al., 2023) against feelings of alienation.

## Discussion

**Figure 1:**
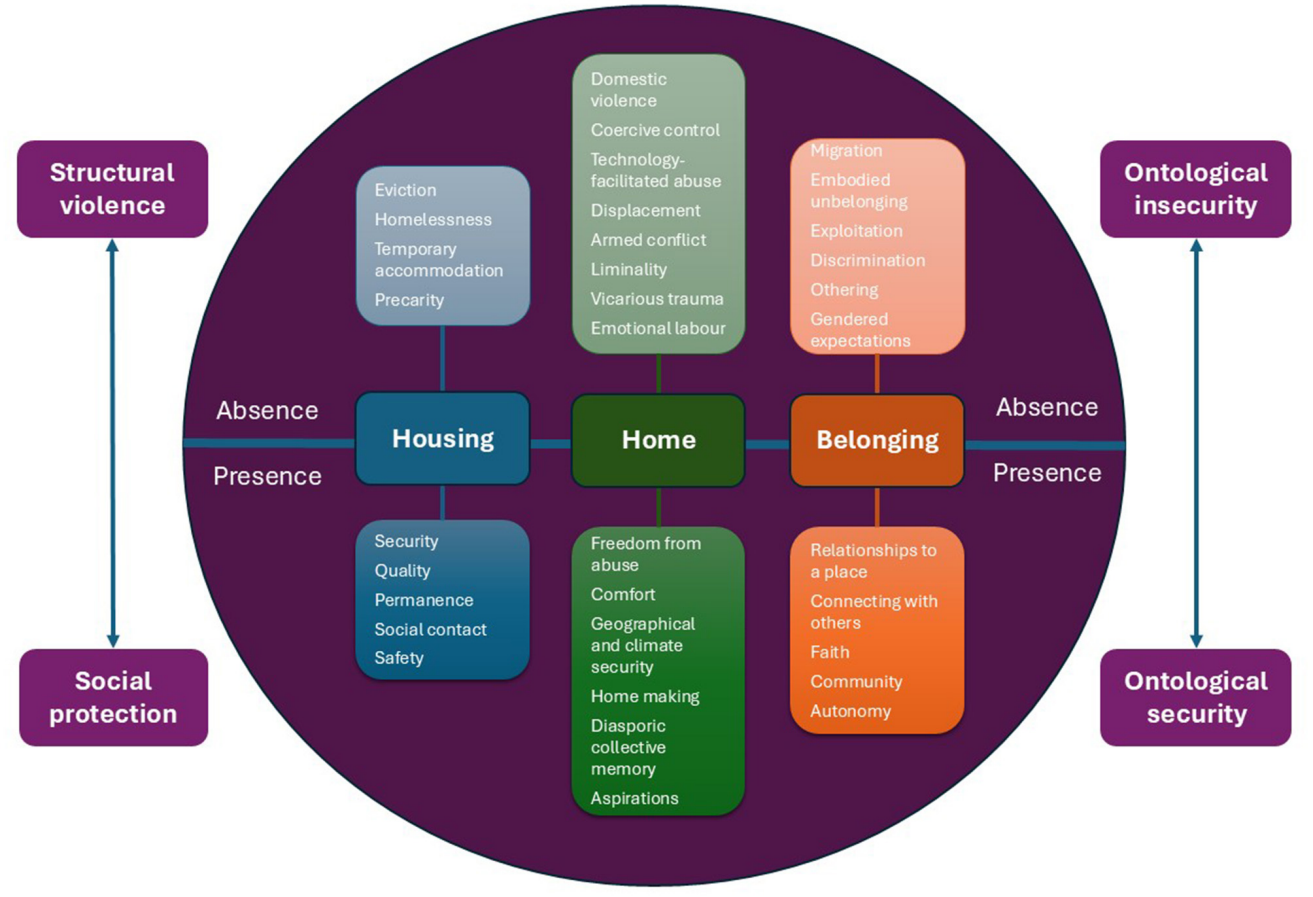
Psychological and structural impacts of the presence or absence of housing, home, and belonging and their relationships to structural violence and ontological security.

A broad, interdisciplinary literature supports the impacts of shelter and place, from housing, through home, to belonging, on women's mental health in diverse contexts (Figure 1). Across the evidence, positive and negative feedback loops recur: the virtuous and vicious cycles described by Farmer et al. (2006). In terms of positive reinforcement, women with social protection often have more stable housing, affording ontological security, predisposing them to better mental health. Those experiencing structural violence often face housing precarity, creating ontological insecurity and increasing their risk of mental health conditions. In terms of negative reinforcement, different forms of structural violence cluster together. For example, increased domestic abuse is associated with climate events (Medzhitova et al., 2023), food insecurity (Jewkes et al., 2023), and armed conflict, each of which is independently linked to precarity.

Despite the compelling evidence that action against structural violence against women is needed, an escalating backlash has caused the stagnation or reversal of programmes addressing gender inequity (UN General Assembly, 2024) in recent years. Policies are being dismantled and reframed, implementation mechanisms undermined, and accountability processes eroded, especially in fragile democracies and countries with anti-democratic governments (UN Women, 2020). Global health funding cuts initiated in 2025 mean that half of women-led and women's rights organizations in humanitarian settings were predicted to close (UN Women, 2025a). Decimation of the sector will substantially diminish the provision of social protections mitigating structural violence, including refuge from domestic abuse, temporary accommodation for displaced persons, group interventions fostering belonging, and gender-focused programming to address patriarchal norms which perpetuate inequity. Based on the evidence surveyed here, widespread cuts to social protection can be expected to exacerbate ontological insecurity, adversely impacting women's mental as well as physical health, and safety.

### Implications for research and practice

Now more than ever, action is needed to center gender equity at the heart of social policy. Complex, non-linear relationships between risk factors for mental health conditions demand multisectoral interventions at all stages of the psychosocial pathways model (Stansfield and Bell, 2019). This includes commitment to tackle distal drivers (gender inequity, stigma, discrimination, economic oppression, punitive laws), alongside proximal needs (interventions addressing women's health, housing, and gender-based violence victimization; WHO OHCHR, 2023). Gender-inclusive polices must be integrated into “shock-proofed” social protection initiatives, to limit the potential for women' rights to be rolled back, following political fluctuations (UN Women, 2025b).

Mental health services need to be tailored for gender differences, including age of onset, clinical presentation, gender roles, sexual, and reproductive health and preventive health needs and trauma histories (Ferrara and Srihari, 2021). Gender responsive, trauma-informed approaches to mental healthcare should be standard practice (Sweeney et al., 2018). Despite a large literature on housing and health, the lack of a gendered perspective hinders the development of interventions responding to these complex, intersectional phenomena (Vásquez-Vera et al., 2022). Currently available evidence suggests that mental health interventions tailored for homeless women are acceptable and effective (Anderson et al., 2024). However, complex social factors, including transient accommodation and IPV can impede rigorous intervention research with marginalized women (Greene et al., 2021). Future research requires sufficient resources to permit longitudinal follow-up of groups who relocate frequently.

Gender-based disadvantage intersects with other aspects of women's identities. The scale of structural violence against minoritised groups requires political will to develop co-produced, holistic services which engage with therapeutic cultural practices, amplify indigenous perspectives on healing, and rebuild trust (Bansal et al., 2022). The importance of place-making offers opportunities to enhance marginalized women's sense of belonging, build trust (Graham-Brown, 2022) and combat isolation (Adlington et al., 2023) by fostering cohesion and facilitating social interaction through community interventions and housing policies. However, interventions furthering ontological security require meaningful action against threats to women's physical and mental health and safety in domestic settings. Political commitment and public health approaches (UN General Assembly, 2019b) are essential to mitigate mental health impacts of IPV across the life course (Oram et al., 2022).

The impacts of wider policy decisions on the social determinants of women's health and health equity require recognition. The assertion that there can be “no health without mental health” (WHO, 2025) recognizes the need to mainstream mental health across government decision-making. A structured approach to policy integration can be taken through resources such as the Mental Wellbeing Impact Assessment Toolkit (National MWIA Collaborative, 2011). Tested in sites across England, the toolkit guides implementers to consider, systematically, how four protective factors (inclusion, participation, control, and community assets) are affected by population characteristics, wider determinants, and social relationships. Implementers are then guided to consider how each protective factor is in turn impacted by aspects of equity and social justice. This approach has been applied to over 1,000 initiatives globally, demonstrating the feasibility of integrating attention to social determinants of mental health into policy making (Coggins, 2025).

However, the cascade of global disinvestment in health research and practice mitigating inequity in 2025 leaves the field in a position of profound uncertainty (Keynejad et al., 2026), while populist anti-migrant sentiment continues to rise. Until recent ideological shifts, the 2030 Agenda for Sustainable Development (UN General Assembly, 2015) and Global Compact for Safe, Orderly and Regular Migration (IOM, 2018) had attracted broad consensus. Abrupt loss of consensus at a time when displaced, traumatized and conflict-affected populations are growing creates conditions threatening to community mental and physical health. Without action, decimation of international aid and research programmes amid multiple extended wars fought in urban settings threaten catastrophic impacts on women, intersectionally minoritised groups, their children and families (Clark, 2025).

## Conclusions

This Perspective presents an integrative sociological framework for understanding the impacts of a continuum of relationships to shelter and place, from housing, through home, to belonging on women's mental health. Drawing on interdisciplinary evidence and grounded in clinical understanding, we show how the concept of structural violence illuminates complexity by identifying positive and negative feedback loops between diverse forms of social prosperity/adversity and mental (ill)health. We demonstrate how ontological (in)security is an instructive organizing principle for the experiences of personal coherence, security and consistency fostered by housing, home and belonging, through examples of harm and resilience in the face of their violations and deprivations. While many distal drivers of structural violence are beyond practitioners' individual influence, the personalized perspective offered by centring ontological security is an essential defense against systems and ideologies which dominate, dehumanize and other. In the current moment of global precarity, we call for a renewed commitment to research and care which dismantles architectures of structural violence, starting with the foundational importance of safe, secure, habitable housing and community environments fostering belonging. Researchers, policy-makers, clinicians, housing providers, voluntary sector professionals, cultural, spiritual and community groups, and people with lived experience must unite to reaffirm the universal rights of all people to the highest attainable standards of health and living.
